# Impacts of patient and family engagement in hospital planning and improvement: qualitative interviews with patient/family advisors and hospital staff

**DOI:** 10.1186/s12913-022-07747-3

**Published:** 2022-03-18

**Authors:** Natalie N Anderson, Kelly Dong, G. Ross Baker, Lesley Moody, Kerseri Scane, Robin Urquhart, Walter P Wodchis, Anna R Gagliardi

**Affiliations:** 1 Toronto General Hospital Research Institute, University Health Network, 200 Elizabeth Street, 13EN-228, M5G2C4 Toronto, Canada; 2 Faculty of Medicine, University of Toronto, Toronto, Canada; 3 Institute of Health Policy, Management and Evaluation, University of Toronto, Toronto, Canada; 4 Princess Margaret Cancer Centre, University Health Network, Toronto, Canada; 5 Patient Partnerships, University Health Network, Toronto, Canada; 6 Department of Community Health and Epidemiology, Dalhousie University, Halifax, Nova Scotia Canada

**Keywords:** Patient engagement, Patient-centred care, Hospitals, Organizational capacity, Hospital planning, Hospital improvement, Interview, Resource allocation

## Abstract

**Background:**

Patient engagement (PE) in hospital planning and improvement is widespread, yet we lack evidence of its impact. We aimed to identify benefits and harms that could be used to assess the impact of hospital PE.

**Methods:**

We interviewed hospital-affiliated persons involved in PE activities using a qualitative descriptive approach and inductive content analysis to derive themes. We interpreted themes by mapping to an existing framework of healthcare performance measures and reported themes with exemplar quotes.

**Results:**

Participants included 38 patient/family advisors, PE managers and clinicians from 9 hospitals (2 < 100 beds, 4 100 + beds, 3 teaching). Benefits of PE activities included 9 impacts on the capacity of hospitals. PE activities involved patient/family advisors and clinicians/staff in developing and spreading new PE processes across hospital units or departments, and those involved became more adept and engaged. PE had beneficial effects on hospital structures/resources, clinician staff functions and processes, patient experience and patient outcomes. A total of 14 beneficial impacts of PE were identified across these domains. Few unintended or harmful impacts were identified: overextended patient/family advisors, patient/family advisor turnover and clinician frustration if PE slowed the pace of planning and improvement.

**Conclusions:**

The 23 self reported impacts were captured in a Framework of Impacts of Patient/Family Engagement on Hospital Planning and Improvement, which can be used by decision-makers to assess and allocate resources to hospital PE, and as the basis for ongoing research on the impacts of hospital PE and how to measure it.

**Supplementary Information:**

The online version contains supplementary material available at 10.1186/s12913-022-07747-3.

## Introduction

Hospitals provide a large proportion of healthcare services and consume the bulk of healthcare spending in many countries worldwide [1]. Despite considerable investment, research shows that hospital quality of care could be improved. For example, a survey of hospital patients across 13 countries showed that overall quality ratings were low to moderate, ranging from 35 to 60% [2]. Another survey of 90,000 + patients hospitalized in Canada similarly revealed moderate views of the care they received: 56% reported that care was well-coordinated and 66% reported being well-informed about their condition and its management [3]. Given the imperative to improve patient experiences and outcomes in a cost-efficient manner, strategies are needed to support quality improvement efforts in hospitals.

Healthcare policy-makers, funders and executives require knowledge about strategies that improve the organization and delivery of hospital care to inform decision-making regarding the allocation of scarce resources. One approach for optimizing the quality and safety of hospital care is to engage patients and family/care partners in planning, evaluating and improving services for the benefit of all patients. In this context, patient engagement (PE) is defined as patients, families or their representatives, and healthcare professionals working in active partnership to improve health and healthcare [4]. This can be achieved in a variety of ways and across a spectrum of engagement that can involve patients or their representatives in single (e.g. questionnaire, focus group) and/or ongoing (e.g. project team, standing committee) activities to plan, deploy, evaluate or improve facilities, programs and care services [5].

Accumulating research offers insight on how to operationalize PE. Two frameworks have been developed to describe PE capacity, referring to essential infrastructure and processes that promote and support PE. An investigation by Baker et al. involving 10 case studies of PE in Canada, the United States and England revealed three processes common to “engagement-capable” organizations: they recruited and prepared patients for engagement, they encouraged and trained staff for patient engagement, and they actively promoted and supported PE [6]. Oostendoorp et al. surveyed patients, clinicians, managers, policy-makers and researchers in 16 countries to generated the Measuring Organizational Readiness for Patient Engagement (MORE) framework comprised of 22 elements of organizational PE capacity in three categories: tasks (e.g. sharing the organizational vision for PE with all employees), resources (e.g. training health professionals in PE) and context (e.g. performance measures include PE) [7]. Evaluation of PE initiatives revealed numerous enablers and barriers, which also provides illuminating guidance for PE. For example, dedicated funding, staff and technology, and organizational commitment and staff champions are needed to facilitate PE [8, 9]. Barriers of PE included uncertainty among patients about their role, resistance from clinicians to working with patients and token PE, resulting in little or no service improvement [10]. A survey of 91 general hospitals revealed that hospitals of various types/sizes achieved an environment conducive to PE by featuring PE in multiple units or departments and employing a greater proportion of PE approaches that involved more than only consulting patients [11].

Despite the accumulating evidence on conditions needed to promote and support PE, little is known about the impacts of PE, knowledge needed by decision-makers to monitor the benefits of PE and allocate the necessary resources [12]. A systematic review (48 studies, 1990–2016) revealed numerous benefits of PE including: improved health care (e.g. new services, greater access to services), new or improved policies (e.g. clinical care models, strategic plans), strengthened governance (e.g. policy audit, culture change) and informational resources (e.g. patient information) [13]. Additional knowledge about the impact of PE is needed to establish a basis for measuring investments in PE and guide decision-making about PE capacity. In particular, input from a range of stakeholders, including involved patients/family can ensure that future measures of PE impact reflect blended perspectives, leading to holistic assessment of PE’s intended goals.

The overall aim of this study was to elaborate on the impacts of PE identified by Bombard et al. by exploring the perspective of multiple stakeholders on the impact of PE specifically in the hospital setting. The objective was to gain insight into the potential impacts of PE as experienced by patient/family advisors, PE managers, clinicians and executives involved in PE for hospital planning and improvement. The resulting identified impacts could be transformed into measures of PE and used in the future by researchers, healthcare policy-makers, hospital executives or PE managers to assess, support and continuously enhance PE. The identified impacts of PE may inform future efforts to identify high value elements of patient engagement that influence patient experience, outcomes, and costs.

## Methods

### Approach

We employed a qualitative research design to explore the impact of hospital PE activities based on the experiences of those involved in different roles [14]. Specifically, we used qualitative description involving semi-structured interviews, which captures participants’ explicit views and experiences, and does not generate or test theory [15]. We complied with standards for reporting qualitative research and enhancing rigor [16, 17]. Ethical approval was obtained from the University Health Network Research Ethics Board. The study was conducted by a team of health services researchers (4), patient research partners with experience of having used hospital services or as hospital patient/family advisors (3), patient engagement managers (2), and a biostatistician (1). Representatives from the Ontario Ministry of Health, Ontario Hospital Association, and Canadian healthcare accreditation agency contributed to the study. All of the research team members including patient research partners helped with the conceptualization of the study and study design (e.g. reviewed interview guide), anddata analysis and result interpretation (reviewed themes and quotes) by taking part in meetings and reviewing material shared with them by email. Written informed consent was obtained prior to interviews and there was no conflict of interest between researchers and participants.

### Sampling and recruitment

We used purposive sampling to recruit individuals who varied by role (managers responsible for PE, patient/family advisors and clinicians involved in at least one PE project), hospital type (< 100 beds, 100 + beds, teaching) and setting (local health integration network [LHIN]). We recruited participants from hospitals with high PE capacity, identified by a prior survey of 91 PE managers about hospital capacity for PE, and described in detail elsewhere. 11 In brief, high PE hospitals were those that featured PE in planning and improvement activities (e.g. developing policies, strategic plans, quality indicators or educational material for patients) across multiple clinical and corporate departments, and a greater degree of collaborationwith (e.g. partnered decision-making via membership on standing committees or project teams) rather than consultation of (e.g. solicit input or feedback via interview, focus group, or questionnaire that may or may not be used in decision-making) patient/family advisors. PE managers from hospitals with high PE capacity were invited to participate via email between January 13, 2020 and July 16, 2020. Interviewed PE managers referred us to patient/family advisors andclinicians (snowball sampling). We aimed to recruit 1 PE manager, 2 patient/family advisors and 2 clinicians from 2 hospitals of each type for a minimum total of 30 interviews. Sampling was concurrent with data collection and analysis, and proceeded until data saturation, when no new themes emerged from further interviews, as established through research team discussion.

### Data collection

We conducted telephone interviews between January 21 and July 16, 2020. NA (MPH, Research Associate) and ARG (PhD, Senior Scientist/Professor) jointly conducted the first two interviews, independently reviewed transcripts, then discussed and refined wording of interview questions. NA subsequently conducted all interviews. We purposefully developed a brief, simple interview guide consisting of two broad, open questions that reflected the study aim of identifying the impact of PE, with no a priori influence from existing models, theories or frameworks. The aforementioned research team reviewed and refined the interview guide (Additional file 1) prior to use. To situate the interview, we first asked participants to describe a PE initiative in which they were involved (findings published elsewhere). We then asked about the impact of PE for this or other initiatives including benefits and unintended consequences. Interviews ranging from 20 to 73 min were audio-recorded and transcribed.

### Data analysis

We used content analysis and constant comparative technique to inductively identify themes [14]. We managed data with Microsoft Office (Word, Excel). NA and ARG independently coded the first two interviews, compared and discussed themes to develop a preliminary codebook of themes and exemplar quotes (first level coding). NA coded subsequent interviews to expand or merge themes (second level coding), conferring with ARG about uncertainties as needed. NA met with ARG on two occasions to review, discuss and refine coding. We tabulated data (themes, quotes) by participant role and hospital type to compare themes. We used summary statistics to describe participant characteristics, and text and tables to describe key themes. The research team reviewed and confirmed themes.

As noted, to plan and monitor PE, there is need for a clearer understanding of measures of PE impact and their relationship. In keeping with the descriptive qualitative approach, we first generated themes with no influence from existing theory (as described above). Then, to further organize and interpret themes and their relationship, we mapped themes to an existing framework conceptualized by Levesque and Sutherland [18]. We chose this framework because it is: current (published in 2020, so based on most recent research), comprehensive (based on 110 performance measures from 19 frameworks distilled into 12 constructs spanning 5 domains: patient needs and expectations, healthcare resources and structures, healthcare functions and processes, receipt and experience of services, and outcomes) and reflects multiple perspectives (integrates patient, clinician and health system leader perspectives on performance assessment). NA and ARG independently mapped themes to the five domains of the framework, then resolved differences through discussion, and the research team reviewed and approved mapping. We depicted impacts using a diagram to display impact measures by category and their potential relationship.

## Results

### Participants

We interviewed 20 patient/family advisors (mean age 66.2 years, 75.0% women), 10 clinicians (1 physician, 6 nurses, 1 social worker, 2 occupational therapists, 90.0% women) and 8 PE managers (mean 10.9 years PE experience, 75.0% women) affiliated with 9 hospitals: 2 < 100 bed (8 participants), 4 100 + bed (21 participants) and 3 teaching (11 participants) hospitals (Table 1).

**Table 1:** Participant characteristics

Role	Affiliation by hospital type			Sub-total
	< 100 beds	100 + beds	Teaching	
PE managers	2	4	2	8
Patient/family advisors	4	10	6	20
Clinicians	2	6	2	10
Sub-total	8	20	10	38

### PE Impacts

Additional file 2 includes themes and quotes reflecting impacts. Themes with select quotes are discussed here. There were no discrepancies in themes by hospital type (< 100 beds, 100 + beds, teaching), and little by role (patient/family advisor, PE managers, clinicians). Themes unique to role are noted in Additional file 2, Tables 2 and 3, and in the following text. We identified impacts at two levels: 9 impacts reflect development of capacity for PE processes and among those involved in PE, and 14 impacts reflect the benefits of PE to hospitals, clinicians/staff and patients/family.

**Table 2:** Impacts of PE on PE capacity and those involved

Theme	Sub-theme	Exemplar quotes	Articulated by:		
			Patients/Family	PE Managers	Clinicians
PE Capacity	New PE approaches or processes developed and widely replicated	And then it was decided that the PE activity process was a success that we’d start to look at doing it for other units (027 PE manager < 100)	X	X	X
Patient/Family Advisors	Satisfaction with contributions that help others	You could see the sense of pride for them as true patient partners because they knew the impact that it was going to have on the families into the NICU (022 clinician 100+) This one [PE activity] was quite meaningful and satisfying for all the patient members who took part… I think we’re all very pleased when we saw the final version; extremely pleased with the work we had done and all contribution. So overall we felt it was a very satisfactory experience (035 patient/family < 100)	X	X	X
	Feeling valued as perspectives truly heard and used	I have always felt that my contribution is valued and listened too. And taken into account when it comes to decision-making (030 patient/family 100+)	X	X	X
	Learning about the complexity of healthcare	The impact on patients is that they understood more what the system is like now and they understood more of what they want out of the system. I think that was a big impact on them (040 clinician 100+) PFA’s really get a good sense of the complexity of trying to offer any service. So they always tend to comment on how much more they appreciate the complexity of the healthcare system having been involved and engaged in, in trying to solve some of these gaps (038 corporate executive teaching)	---	X	X
	Feeling empowered leading to greater engagement	I think the other impact on some of the patients and families that we dealt with was empowerment. They felt empowered to be able to speak up and provide their feedback… they really became more and more engaged as time went on (040 clinicians 100+)	---	X	X
	**Unintended**: Overburdened by frequent deployment	Tiring them [patient/family advisors] out, asking too much, having to do too much when they may still be caring for a loved one that’s sick (001 PE manager < 100) One of the things that we hear about is that sometimes we might over reach individuals. So patients mention that they might be feeling over contacted, right? So I think that is a bit of a risk; they may feel burdened (031 clinician 100+)	X	X	---
	**Unintended**: Conflicting expectations lead to turnover	A lot of people that have come on board do not really have positive experiences, and that’s why they want to get involved. To be able to put your own issues aside and look at the greater good, some people really struggle with that and we’ve had people leave just because of that (018 patient/family 100+)	X	X	X
Staff involved in PE	Reminder of importance of listening to patients	It reinforces what really matters when it comes to healthcare and the importance of listening to patients. The importance of the patient voice; and when I say the importance of teamwork, it’s not just teamwork with staff; it’s teamwork with patients, teamwork with their families (015 patient/family teaching)	---	X	X
	Reminder of why they chose a healthcare career	I think for staff it’s a reminder of why they went into healthcare and the importance of teamwork … I think it reinforces why they went into healthcare (015 patient/family teaching) It guides clinicians and staff to really think about the reason why they came to working in healthcare (028 PE manager teaching)	X	X	---
	Greater appreciation of PE for planning and improvement	The finished product looked quite a lot different as a consequence of patient input and feedback. So I think those involved saw that as really quite useful and a little bit eye-opening. You know I think to a degree it opened people’s eyes that patients can have some pretty useful things to say that can actually change what we do (012 clinician teaching) I think its increased awareness around patient engagement in staff. We believe strongly enough in this process that we take the feedback that they give us which is very valuable and staff can see that. So I think it increased awareness of how important it really is (027 PE manager < 100)	X	---	X
	Increased openness or willingness to engage patients	Now, everybody’s mad if the patient isn’t at the committee. They’re, like, where’s the patient? Can we have the meeting without them? (001 PE manager < 100)	X	---	---
	**Unintended**: Frustrated if PE slows pace	Patients always slow down the process of moving towards a goal and making decisions because there’s a multitude of questions and you’re dealing with a number of people who are inexperienced … it’s the cost of doing business with patients and I’m sure that some of our professionals are frustrated by that (039 patient/family teaching)	X	---	---

**Table 3:** Impacts of PE on hospitals, clinicians/staff and patients/family

Theme	Sub-theme	Exemplar quote	Articulated by:		
			Patients/Family	PE Managers	Clinicians
Hospital structures and resources	New or improved hospital policies and strategic plans	At times, we have to put males and females together in rooms when hospital occupancy is high… So we created policies and information to share with patients around that (001 PE manager <100) We’ve also engaged patient and family feedback through our surveys and our patient relations data because we get some great level of data and information that we can leverage to build our quality improvement plan every year (032 corporate executive 100+)	X	X	X
	New or improved facilities, programs and services	We had an issue where the public phone was in a bad place. So we just brought it forth and it was changed. It was put in a better area (002 patient/family <100) I think the impact was that people actually did get healthier food (003 patient/family teaching) For sure there was a lot more consistency in terms of the services that were provided and the level of service as well as the kind of service provided (021 clinician 100+) So we were a pilot site for the Bundle Care Program … They [patient/family advisors] were helping to inform what then became our future state pathway which we have really continued to enact today (025 clinician 100+)	---	X	X
	Resources for patients/family (e.g. discharge information, educational material)	We have seen a significant improvement in the clarity of the consent letters that our patients are being asked to sign to be part of a research project (017 patient/family 100+) It [discharge information sheets] was successful with the emergency department. So anything that can support your education upon discharge from the hospital will prevent hopefully any unnecessary visits; certainly support on-going quality improvement to the care you can provide at home (027 PE manager <100) The resources were utilized… feedback from our social workers who were meeting with clients regularly who had that exposure to those resources [psycho-education material] in the waiting room said that they thought it was great to have those resources available to them while they were waiting (034 clinician 100+)	X	X	X
Clinician or staff functions and processes	Greater work enjoyment	We found that the charge nurses and also the nursing staff were able to achieve a bit more joy in their work (024 PE manager 100+)	X	X	---
	Satisfaction with new or improved facilities, programs or services	I think they [staff] were very happy… So I think they were welcoming that change (013 patient/family 100+) Increased [staff] satisfaction that patients are having somebody to engage with versus just in their rooms when they’re attending with other patients (037 clinician <100)	---	X	X
	Greater ease in fulfilling job requirements	It makes their [staff] job easier (013 patient/family 100+)	---	X	---
	Greater efficiency in healthcare delivery	So the staff would say that it’s a very efficient way, a more efficient way to do their care and that they are getting just as good outcomes with this change in model (025 clinician 100+)	---	---	X
	Greater confidence in information they provided to patients	They [staff] would also feel more confident in the information that they’re giving out to patients and families; that there would perhaps be an increased understanding (027 PE manager <100)	X	---	---
	Improved patient-staff communication	The process was designed to improve communication and to allow much, much better access to the physician group or the residents, and to allied health people because they were on the floor. It really improved communication (007 patient/family teaching) We also surveyed both doctors and nurses … there had been a substantial improvement in the efficiency and quality of communication with patients… nurses have said, we get less questions from families now (012 clinician teaching)	X	X	X
Patient experience	Reassurance that hospital addresses what matters to patients	I think it was reassuring for them [patients] to know that what they felt and what mattered to them in their care was going to be captured and was going to be rolled out with staff and form the basis of what their care would look like. And so I think for patients it was very reassuring; that their needs were being taken very seriously (015 patient/family teaching) Patients knew] that staff at the hospital were listening to what really mattered to them and humanizing them (015 patient/family teaching)	X	X	X
	Increased satisfaction with facilities, programs and services	And it’s a substantial supper and a lighter lunch and inpatients seem to be very pleased with the transition (002 patient/family <100) So the patient satisfaction increases greatly from this [post-discharge contact program] (031 clinician 100+)	---	X	X
	Improved healthcare experience	Patients would say that their experiences are better following the changes that were made (025 clinician 100+)	---	X	X
	Greater understanding of hospital instructions (due to new/ improved resources)	So I think it [patient admission handbook] had a significant impact on patient admission as far as preparation and simplifying the process somewhat for the hospital; people are going in prepared with some knowledge (023 patient/family 100+) The discharge information sheets improved understanding for our patients and families… we could see how it provided clarity for the patient (027 PE manager <100)	X	X	X
Patient outcomes	Decreased wait times	So there’s been a significant reduction in the number of wait time hours between the Emergency Department to in-patient units (011 PE manager 100+)	---	X	---
	Decreased falls	Decreased risk for falls; we did see a decrease in falls (037 clinician <100)	---	---	X
	Decreased readmissions	I’d say, we also saw a reduction in patient readmission rates as well (021 clinician 100+)	---	---	X
	Increased safety	They’re rolling out elements of the model of care … So they’re spreading it unit by unit improving quality, safety, patient experience (011 PE manager 100+)	---	X	---

### Impacts on PE capacity and those involved

Table 2 summarizes themes and exemplar quotes representing 9 impacts of PE on PE capacity and those involved. Beneficial impacts were categorized as impacts on PE capacity, patient/family advisors, and staff involved in PE.

PE activities contributed to increased hospital capacity for PE. As new PE approaches or processes were developed and used, they were then replicated in other units or departments, expanding capacity for PE. For instance, a PE manager stated “and then it was decided that the PE activity process was a success that we’d start to look at doing it for other units”. This view was articulated by patients/family, PE managers, and clinicians.

PE affected patient/family advisors with four beneficial impacts. Patient/family advisors gained a great deal of satisfaction by contributing to PE because they believed that it helped others. Patient/family stated that “I think we’re all very pleased when we saw the final version; extremely pleased with the work we had done and all contribution”. Patient/family advisors felt valued because their perspectives were used to plan or improve hospital facilities, programs or services. PE managers and clinicians said that PE benefited patient/family advisors in two ways: (1) they learned about the complexity of health care to better understand what they wanted from the healthcare system, and (2) they were “empowered to be able to speak up and provide their feedback… they really became more and more engaged as time went on”.

PE affected clinicians/staff with four beneficial impacts. Involvement in PE activities served to remind staff about the importance of listening to patients as a means of improving healthcare services, and reinforced to staff why they chose a healthcare career. It also prompted awareness and appreciation of the importance of PE in hospital planning and improvement. “I think to a degree it opened people’s eyes that patients can have some pretty useful things to say that can actually change what we do” was articulated by a clinician. Patient/family advisors thought that involvement in PE caused staff to be even more open or willing to participate in PE.

Participants identified some potentially unintended consequences. With respect to patient/family advisors, some became overburdened through frequent deployment, while others left the role after onboarding due to conflicting expectations: patient/family advisors articulated opinions based on having had poor experiences but felt dismissed when their concerns were not considered versus healthcare professionals viewing their concerns as biased and not contributing to the greater good. With respect to clinicians/staff, patient/family advisors noted that professionals became frustrated when patient/family advisor questions and inexperience slowed the planning or improvement process.

### Impacts on hospitals, patients/family, clinicians/staff

Table 3 summarizes themes and exemplar quotes representing 14 impacts of PE on hospitals, patients/family and clinicians/staff. Participants said that PE resulted in beneficial impacts on hospital structures and resources, clinician/staff functions and processes, and patient experience and outcomes.

PE resulted in impacts on hospital structures and resources. These included new policies or strategic plans, and improved facilities, programs or services. PE also resulted in resources for patients such as educational material or discharge information. A clinician gave an example: “They [patient/family advisors] were helping to inform what then became our future state pathway which we have really continued to enact today”.

PE resulted in several impacts on clinician/staff functions and processes. These included greater work enjoyment, ease in fulfilling job requirements (articulated only by PE managers) and efficiency in healthcare delivery (articulated only by clinicians); and satisfaction with new or improved facilities, programs and services. Clinicians/staff also had greater confidence in the information they provided to patients (articulated only by patient/family advisors) and experienced improved communication with patients.

As a result of improvements informed by patient/family advisors, participants thought that PE resulted in improved experiences among patients using hospital facilities and services. Patients felt reassured based on knowing that the hospital addresses what matters to patients, and satisfied with new or improved facilities, programs and services. Patients knew that “staff at hospital were listening to what really mattered to them and humanizing them”. Patients reported improved hospital experiences and greater understanding of hospital instructions (such as patient admission information, discharge information) due to new or improved patient information resources. A PE manager felt that “the discharge information sheets improved understanding for our patients and families… we could see how it provided clarity for the patient”.

PE managers and clinicians said that PE resulted in measurably improved patient outcomes such as decreased wait times, falls and readmissions, which overall increased quality and safety.

### Summary of PE impacts

**Fig. 1:**
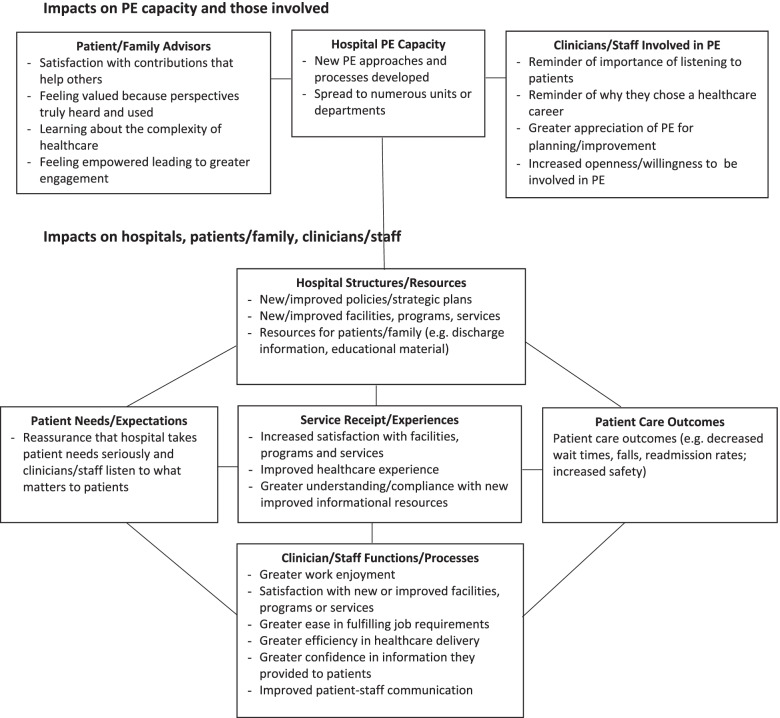
Impacts of Patient/Family Engagement on Hospital Planning and Improvement

Guided by the framework developed by Levesque and Sutherland, we mapped findings to categories of impacts to depict their potential relationships [18]. Fig. 1 summarizes the impacts of PE and suggests a relationship between the impacts on PE capacity and on those involved, and the various impacts of PE on the hospital, clinicians/staff and patients/family. This framework can be used by various stakeholders to identify measures for assessing how well PE is implemented and functioning, and the impact of PE.

## Discussion

Through interviews with 38 patient/family advisors, PE managers and clinicians involved in PE for hospital planning and improvement, we identified a wide range of beneficial impacts of PE. Benefits of PE activities included 9 impacts on hospital PE capacity and involved patient/family advisors and clinicians/staff, such that new PE processes were developed and spread across hospital units or departments, and those involved became more adept and engaged. Benefits of PE outputs to hospitals, patients/family and clinicians/staff included 14 impacts categorized according to an established framework of healthcare performance measurement domains as Patient Needs/Expectations, Hospital Structures/Resources, Clinician/Staff Functions/Processes, Service Receipt/Experience and Patient Care Outcomes. Few unintended or harmful impacts were identified: overextended patient/family advisors, patient/family advisor turnover and clinician frustration if PE slowed the pace of planning and improvement.

Prior research largely addressed PE for purposes other than hospital planning and improvement. For example, a systematic review identified the benefits and challenges of PE in prioritizing research questions or designing research studies [19]. Other studies examined the impact of PE in their own care on clinical outcomes [20, 21]. For example, greater PE of patients with chronic conditions was associated with greater patient adherence to prescription refills, attendance at scheduled visits and immunization [22]. Another body of research focused on measures by which to monitor and improve quality of care. Specific to hospitals, a systematic review of hospital quality indicators identified a total of 248 indicators related to infection, safety, quality and mortality organized as 29 indicators of structure, 122 as process and 97 as outcomes across 10 disease groups [23]. Our study is unique from this prior research on hospital quality measures because we focused specifically on the impact of PE rather than the impact of clinical care or other approaches to quality improvement. To date, Bombard et al.’s seminal review of engaging patients to improve quality of care identified four key impacts of PE: new or improved policies or strategic plans, educational resources for patients, enhanced governance processes and enhanced service delivery [13]. Our results confirm and elaborate on Bombard et al.’s finding. We too found that PE resulted in the same key benefits at the hospital level. We identified additional categories of hospital-level impacts as a result of PE outputs including benefits for clinician/staff functions and processes, enhanced patient experience and improved patient care outcomes. In addition, at the PE level, we identified several positive impacts of PE activities on hospital capacity for PE, and on those involved including patient/family advisors and clinicians/staff.

The findings of this study have several implications for policy, practice and research. Healthcare systems and hospitals routinely monitor performance to publicly report on quality and safety, and to demonstrate eligibility for value-based funding [24]. Although PE in planning and improvement is widespread, to date the only measure used by hospitals to report PE activity was presence of a patient/family advisory committee [25], a measure with limited utility because research shows that PE can be token [10] As a concrete knowledge output, components of the Impacts of Patient/Family Engagement on Hospital Planning and Improvement Framework (Fig. 1) can be transformed by decision-makers such as healthcare policy-makers and hospital leaders into multiple PE performance measures, and used to monitor or evaluate the operationalization and impact of PE. Examples of PE performance measures at the PE activity level include satisfaction among involved patient/family advisors and clinicians/staff with the PE process, and number of units or departments featuring PE activity. Examples reflecting the impact of PE outputs include the number of new or improved policies, strategic plans, facilities, programs, services or patient/family informational material; belief among patients that the hospital cares about what matters to patients; patient healthcare experience; clinician/staff work-life satisfaction and patient care outcomes. Doing so may reveal where investment is needed to support PE and enable the multiple beneficial impacts of PE.

While this study identified only a few unintended impacts (patient/family advisor burden and turnover; clinician frustration with slow pace), they warrant discussion. Prior research showed uncertainty among patients about their role and resistance from clinicians to working with patients, resulting in token PE and little or no service improvement [10]. A survey of hospitals about capacity for PE in planning and improvement revealed that most respondents did not have funding dedicated to PE, perhaps partially explaining these challenges and unintended consequences [11]. However, interviews with representatives from those same surveyed hospitals revealed numerous strategies they employed to overcome such barriers. For example, assembling a large pool of diverse patient/family advisors, matching patients to projects, training patients and health-care workers, involving a critical volume of patients, requiring at least one patient for quorum, asking involved patients to review outputs, linking PE with the Board of Directors, championing PE by managers, staff and committee/team chairs, orientation to PE for new and existing staff, and continuous evaluation and improvement of PE [26, 27]. Hence, even hospitals with little or no dedicated resources can optimize PE capacity in a variety of ways so that patient advisors feel valued and staff feel supported. Still, further investigation is needed to generate insight on how to best utilize patient/family advisors and balance PE with the burden placed on them.

Ongoing research is needed to build on our findings. To date, hospitals have largely assessed performance by relying on measures of patient care outcomes such as infection, readmission and mortality rates [23]. In part, hospitals rely on such patient care outcomes because they are readily measurable with routinely-collected administrative data. To measure impacts revealed by this study, hospitals may need to collect primary data or begin routinely collecting data specific to these measures; for example, satisfaction with the experience among involved patient/family advisors and clinicians/staff with the PE process. For other impacts, hospitals may wish to identify and use existing instruments; for example, Quality from the Patients’ Perspective or 10-item Job Satisfaction Scale for clinicians [28, 29]. Furthermore, given that the PE impacts revealed by this study were either putative or self-reported, future primary research is needed to systematically document changes or improvements prompted by PE, or measure the impact of PE using before-after, time series or other research designs.

Strengths of this research included rigorous methodology based on previous guidelines in collection and reporting of qualitative data [14–17] An existing framework of healthcare performance measures helped organize the results [18] Multiple stakeholders in patient engagement were involved in the research design and process who had expertise in PE. A diverse range of participants were interviewed as well, from individual patients/family advisors and clinicians to PE managers and corporate executives at an organizational level. However, there remain several limitations to this study. Patient/family advisors were largely retired Caucasian women, thus the views expressed may not represent patient/family advisors with diverse characteristics. Additionally, as all of the participants were recruited from hospitals within one LHIN, these findings may not be universally generalizable, depending on the health care system or PE practices.

## Conclusions

Although PE in healthcare planning and improvement is widespread and intuitively important, we lack evidence of its concrete impact. Our study confirmed these impacts in the hospital PE context, and revealed many others including impacts of PE activity on hospital, patient/family advisor and clinician/staff capacity to undertake PE, and impacts of PE outputs on clinician/staff function and processes, patient experience, and patient care outcomes. While these impacts were self-reported by the 38 participants of our qualitative interviews, including patient/family advisors, PE managers and clinicians, the 24 impacts were captured in a Framework of Impacts of Patient/Family Engagement on Hospital Planning and Improvement. The Framework can be used by decision-makers to assess and allocate resources to hospital PE, and as the basis for ongoing research on the impacts of hospital PE and how to measure it.

## Supplementary Information

**Additional file 1.**

**Additional file 2.**

## Data Availability

All data generated or analysed during this study are included in this published article and its supplementary information files.

## Abbreviations

PE: patient engagement
LHIN: local health integration network
