# Supplementary material for: Impacts of patient and family engagement in hospital planning and improvement: qualitative interviews with patient/family advisors and hospital staff
Source: BMC Health Serv Res. 2022 Mar 18;22:360. doi: 10.1186/s12913-022-07747-3 (PMC8932199; doi:10.1186/s12913-022-07747-3)
Supplement: Supplementary file 1 — Additional file 1. [file 12913_2022_7747_MOESM1_ESM.docx]

**Additional file 1. Interview guide**

| Question | Prompts (selectively applied depending on response to questions) |
| --- | --- |
| Please describe a planning or improvement activity that engaged patients in some way that you were involved in or are aware of | - What was the goal of the activity? - What was the intent of engaging one or more patients? - What units/departments were involved in the activity? - Who led or championed the initiative? - Over what period of time did this activity take place? - What was your specific role? |
| What was the impact of the patient engagement activity? | - Was patient input actually used? - Were changes or improvements implemented? - How were services or facilities improved? - If already implemented, how did the change/improvement to facilities/services improve:   - Patient satisfaction or experience?   - Patient knowledge or behavior?   - Staff satisfaction, work-life, or behavior? - Future policies or processes for identifying or engaging patients? - What was the impact of engaging patients/family in the activity? |
| Were there any negative impacts or unintended consequences from the patient engagement? | - Were there competing priorities (time-constraints for patients/clinicians, resources used to PE rather than another activity, competition between departments or units for PE activity/resources) - Was a lack of desire for future patient engagement a result of this activity? - Were there any consequences to patient or staff feelings or actions? - Did any patients withdraw or no longer wish to participate? - Was there decreased organizational enthusiasm? |
