# Supplementary material for: Impacts of patient and family engagement in hospital planning and improvement: qualitative interviews with patient/family advisors and hospital staff
Source: BMC Health Serv Res. 2022 Mar 18;22:360. doi: 10.1186/s12913-022-07747-3 (PMC8932199; doi:10.1186/s12913-022-07747-3)
Supplement: Supplementary file 2 — Additional file 2. [file 12913_2022_7747_MOESM2_ESM.docx]

**Additional file 2. Themes and quotes on the impact of PE from multiple perspectives**

**Impacts on PE capacity and those involved**

| **Theme** | **PE Managers** | **Patient/family advisors** | **Clinicians** |
| --- | --- | --- | --- |
| PE Capacity | New PE approaches/processes widely replicated   - We’ve been selected a few different times to do presentations on the methodology we used as its been seen as being a productive or successful way of partnering with patients and families… We are beginning our hospital-wide way-finding strategy and the same methodology that was used for the development of our declaration will be used to create the engagement forum for our way-finding strategy with patients and families (010 PE manager 100+) - And then it was decided that the PE activity process was a success that we’d start to look at doing it for other units (027 PE manager <100) - I think the impact now is that it’s [patient declaration of values] starting to be used for how people are going to partner with patients in their care. It’s being used at the driver for the foundation for different projects for discussions within the quality and safety… And so its providing direction in a different policy now (028 PE manager teaching) | New PE approaches/processes widely replicated   - And then it made sense that the people that were involved in doing those calls would get involved in the next step with the improvement plan calls as well (18 pat 100+) - It has sort of extended to different areas after that; there’s other things that people expressed an interest in and gone on discussing with them in different forums (023 patient/family 100+) | New PE approaches/processes widely replicated   - We’re also going to be doing something similar; we have some communication boards in our birthing unit and we’re updating those and we’re going to follow the same process with speaking to the mom’s to ask them what kind of information should be there. How can they be involved and putting information on the boards, etc. (008 clinician teaching) - We’ve been involved in a lot of the Ontario Health team work within the last year and I know we also engaged some of the same patient and family advisors in our OHT work and they were able to bring you know what we learned in the ISM project to some of the OH team work that we’ve been involved in. Really thinking about what really worked with the ISM project, what didn’t work and then bringing those things and surfacing those barriers to part of the OHT work just so that we’re not doing the same old, right? (021 clinician 100+) - So it’s been something that we’re actually hoping through our strategic goals to spread across all discharge patients (031 clinician 100+) |
| Patient/Family Advisors | Satisfaction with contributions that help others   - The other impact is that we received 100% in terms of feedback with those patient and family advisors that were engaged in this process around their satisfaction and felt that their voices and perspectives were heard, that’s really, really important to us (010 PE manager 100+)   Feeling valued because perspectives truly heard and used   - They [patient/family advisors] felt welcomed and they felt valued in my opinion (011 PE manager 100+) - I think that they feel heard and listened to (027 PE manager <100)   Unintended:  Overburdened by frequent deployment   - Tiring them [patient/family advisors] out, asking too much, having to do too much when they may still be caring for a loved one that’s sick (001 PE manager <100)   Unintended:  Conflicting expectations leads to turnovers   - It’s not a role for everybody let me just put it that way because we’ve had some people come and they just didn’t like it at all because it isn’t like you’re working directly with patients all the time, it’s a lot of meetings and things like that (018 patient/family 100+) | Satisfaction with contributions that help others   - I really do feel proud to be doing this kind of work and honoured to be doing it and I think it is because you see the benefit and you see the commitment (015 patient/family teaching) - It was a wonderful experience because our voices were definitely being heard. We were engaged. We were listened too and it was really a wonderful experience (019 patient/family 100+) - I’m honoured to be able to sit on some of these committees with them and to have my say on perhaps changing things for the better in the hospital. I just feel honoured that I can be a part of it... I’m seeing some of the end results of things that we’ve been working on and how they’ve become to fruition (026 patient/family 100+) - This one [PE activity] was quite meaningful and satisfying for all the patient members who took part… I think we’re all very pleased when we saw the final version; extremely pleased with the work we had done and all contribution. So overall we felt it was a very satisfactory experience (035 patient/family <100)   Feeling valued because perspectives truly heard and used   - Well I just think it’s a wonderful opportunity when you’re in a hospital environment to know that you’re there representing a voice that maybe people don’t even know that they have sometimes. And I just feel like it’s a win, win. It’s a win for us as advisors because we feel valued and we have seen some of the things that we’ve suggested for change. And we feel that that enhances delivery as well (019 patient/family 100+) - I think everyone who participated felt heard and valued and it was worth their contribution (029 patient/family 100+) - I have always felt that my contribution is valued and listened too. And taken into account when it comes to decision-making (030 patient/family 100+) - In our most recent meetings it was made clear to us that the hospital really valued the contribution of the PFAC members… we have been reassured over and over that our contributions to the hospital is extremely valuable. So we are very valued and we’re reminded about this over and over. So I think we are doing a worthwhile job as PFAC members (035 patient/family <100   Learning about the complexity of healthcare   - It’s really a gratifying experience because you always get patients on the committee that are interested in the health system and they educate us and we understand what’s going on (003 patient/family teaching) - I learn something about hospital programs, about people individually and about how I can make things better (030 patient/family 100+)   Feeling empowered leading to greater engagement   - Now I have the opportunity to go to other hospitals and share what I’ve learnt and to learn from them. I appreciate that <hospital name> is passionate about creating that community of learning in the broader network (029 patient/family 100+)   Unintended:  Overburdened by frequent deployment   - One of the things that we hear about is that sometimes we might over reach individuals. So patients mention that they might be feeling over contacted, right? So I think that is a bit of a risk; they may feel burdened (031 clinician 100+)   Unintended:  Once onboarded, don’t enjoy role and leave   - A lot of people that have come on board do not really have positive experiences, and that’s why they want to get involved. To be able to put your own issues aside and look at the greater good, some people really struggle with that and we’ve had people leave just because of that (018 patient/family 100+) | Satisfaction with contributions that help others   - You could see the sense of pride for them as true patient partners because they knew the impact that it was going to have on the families into the NICU (022 clinician 100+)   Feeling valued because perspectives truly heard and used   - I think they [patient/family advisors] felt valued that we were caring about their experiences (025 clinician 100+)   Learning about the complexity of healthcare   - The impact on patients is that they understood more what the system is like now and they understood more of what they want out of the system. I think that was a big impact on them (040 clinician 100+)   Feeling empowered leading to greater engagement   - The impact on our committee members and volunteers was that they continue to be engaged in the hospital (037 clinician <100) - I think the other impact on some of the patients and families that we dealt with was empowerment. They felt empowered to be able to speak up and provide their feedback... they really became more and more engaged as time went on (040 clinicians 100+)   Unintended:  Once onboarded, don’t enjoy role and leave   - So near the end of the activity we started to lose some patient and family partners (040 clinician 100+) |
| Clinicians and Staff Involved in PE | Reminder of why they chose a healthcare career   - It guides clinicians and staff to really think about the reason why they came to working in healthcare (028 PE manager teaching)   Greater appreciation of PE for planning/improvement   - I think its increased awareness around patient engagement in staff. We believe strongly enough in this process that we take the feedback that they give us which is very valuable and staff can see that. So I think it increased awareness of how important it really is (027 PE manager <100)   Increased openness/willingness to engage patients   - Now, everybody’s mad if the patient isn’t at the committee. They’re, like, where’s the patient? Can we have the meeting without them? (001 PE manager <100) - I think that one of the worries that we had was around ensuring that we were getting representative patient voices. It was just a constant worry that we were developing something that was representative of everyone as best we could (028 PE manager teaching)   Unintended:  Frustrated if PE slows pace   - Patients always slow down the process of moving towards a goal and making decisions because there’s a multitude of questions and you’re dealing with a number of people who are inexperienced … it’s the cost of doing business with patients and I’m sure that some of our professionals are frustrated by that (039 patient/family teaching) | Reminder of importance of listening to patients   - It reinforces what really matters when it comes to healthcare and the importance of listening to patients. The importance of the patient voice; and when I say the importance of teamwork, it’s not just teamwork with staff; it’s teamwork with patients, teamwork with their families (015 patient/family teaching)   Reminder of why they chose a healthcare career   - I think for staff it’s a reminder of why they went into healthcare and the importance of teamwork … I think it reinforces why they went into healthcare (015 patient/family teaching) | Reminder of importance of listening to patients   - In terms of just sending a clear signal to the staff that we are absolutely here and designing with our patients from that perspective, I think that was an important good impact (008 clinician teaching)   Greater appreciation of PE for planning/improvement   - The finished product looked quite a lot different as a consequence of patient input and feedback. So I think those involved saw that as really quite useful and a little bit eye-opening. You know I think to a degree it opened people’s eyes that patients can have some pretty useful things to say that can actually change what we do (012 clinician teaching) |

**Impacts on hospitals, patients/family and staff**

| **Theme** | **PE Managers** | **Patient/family advisors** | **Clinicians** |
| --- | --- | --- | --- |
| Hospital structures/  resources | New/improved hospital policies/strategic plans   - At times, we have to put males and females together in rooms when hospital occupancy is high… So we created policies and information to share with patients around that (001 PE manager <100) - The Patient Partners played a great role in our communication plan around no visitors policy and the rationale for no visitors. Everyone understood, it’s not what we want to do but they’re the ones that pushed us to think, okay well what about iPads and virtual visits and we ramped that up just as quickly (020 PE manager 100+) - Our patient relations staff were actually being called to the unit a lot more than they had before and they were being engaged in issues very early on and able to solve them while the patient was still here (024 PE manager 100+)   Resources for patients/family (e.g. discharge information, educational material)   - It [discharge information sheets] was successful with the emergency department. So anything that can support your education upon discharge from the hospital will prevent hopefully any unnecessary visits; certainly support on-going quality improvement to the care you can provide at home (027 PE manager <100) | New/improved hospital policies/strategic plans   - Now each of the units are actually identifying one of the things that they’re working on and how that links to the strategic plan… It has been launched and now each of the programs are doing their own goals and tying it to the strategic plan (005 patient/family teaching) - I think it [Patient Declaration of Values] serves as a guideline of what our role is, what we are representing; that these are the things that matter to patients and we need to make sure that when these higher level discussions are going on around patients and incidence; that the focus remains on that patient. It doesn’t become too clinical. It stays human (015 patient/family teaching)   New/improved facilities, programs and services   - So now a dietary staff member meets every active care patient every day to try and enable a solid menu plan for that person, we figure it’s enhancing their care and will enable their betterment (002 patient/family <100) - A lot of our patients were coming into our ER department and they were there waiting to go in, they’d be knocking on the door; could we have a drink of water? Well, there is no reason then, that you couldn’t have proper water dispensing area where it’s required most, right? So we talked about it, it meant that they had to change some of the plumbing and run some lines and do this and that but guess what, it’s there (002 patient/family <100) - We had an issue where the public phone was in a bad place. So we just brought it forth and it was changed. It was put in a better area (002 patient/family <100) - They are getting their big meal in the evening. It is also coming half an hour later; it used to be a 4:30 pm meal, [now] it’s a 5 o’clock meal (002 patient/family <100) - I think the impact was that people actually did get healthier food (003 patient/family teaching) - So it was an improvement in the procedure because now the papers [care plans] can’t get lost and it just comes up right on the patients file (013 patient/family 100+)   Resources for patients/family (e.g. discharge information, educational material)   - The materials for the testing centre, the unique stand-alone testing centre, I was told they were implemented and they were used (014 patient/family teaching) - The materials for <hospital name> advising patients that they could no longer bring family into the hospital with them; those were used (014 patient/family teaching) - We have seen a significant improvement in the clarity of the consent letters that our patients are being asked to sign to be part of a research project (017 patient/family 100+) | New/improved hospital policies/strategic plans   - We pulled together a team to develop and refresh our seniors care framework within the organization. Within the forums they [patient/family advisors] were able to help us develop our strategy for seniors care within the organization (021 clinician 100+)   New/improved facilities, programs and services   - For sure there was a lot more consistency in terms of the services that were provided and the level of service as well as the kind of service provided (021 clinician 100+) - So we were a pilot site for the Bundle Care Program … They [patient/family advisors] were helping to inform what then became our future state pathway which we have really continued to enact today (025 clinician 100+) - We had a dialysis patient who talked about that whenever she came in for her dialysis, it was just so cold in the area. She was willing to talk to a leader from facilities about this and she actually came on site and they toured the area together. And by having that direct leader to patient contact, it made a great change and they were able to warm up the area once and for all (031 clinician 100+)   Resources for patients/family (e.g. discharge information, educational material)   - The resources were utilized… feedback from our social workers who were meeting with clients regularly who had that exposure to those resources [psycho-education material] in the waiting room said that they thought it was great to have those resources available to them while they were waiting (034 clinician 100+) |
| Clinician/staff functions and processes | Greater work enjoyment   - We found that the charge nurses and also the nursing staff were able to achieve a bit more joy in their work (024 PE manager 100+)   Greater confidence in information they provided to patients   - They [staff] would also feel more confident in the information that they’re giving out to patients and families; that there would perhaps be an increased understanding (027 PE manager <100)   Improved patient-staff communication   - Now that patients sit on our committees and are much more part of the decisions that we make and the things that we do, everybody wants to come and present at PFAC when they’re doing something new because, they’re like, we want to hear if the patients like it (001 PE manager <100) | Greater work enjoyment   - The residents were saying that because they now know where things are on the unit, they’re a lot less frustrated. They’re not looking for things all the time because that unit is somewhere they are all the time… it’s so much easier (007 patient/family teaching)   Satisfaction with new or improved facilities, programs or services   - I think they [staff] were very happy… So I think they were welcoming that change (013 patient/family 100+)   Greater ease in fulfilling job requirements   - It makes their [staff] job easier (013 patient/family 100+) - I would hope that they felt that we were trying to help; to make their job easier and better (014 patient/family teaching)   Improved patient-staff communication   - The process was designed to improve communication and to allow much, much better access to the physician group or the residents, and to allied health people because they were on the floor. It really improved communication (007 patient/family teaching) - I think it’s a more open process now and I find that they [staff] are more willing to bring us stuff to work on (036 patient/family <100) | Satisfaction with new or improved facilities, programs or services   - We have staff satisfaction rates of about 90% (006 clinician <100) - The NICU staff love it. The staff loved the tool (022 clinician 100+) - So in talking with staff right; they feel that the changes have been for the better (025 clinician 100+) - Increased [staff] satisfaction that patients are having somebody to engage with versus just in their rooms when they’re attending with other patients (037 clinician <100)   Greater efficiency in healthcare delivery   - So the staff would say that it’s a very efficient way, a more efficient way to do their care and that they are getting just as good outcomes with this change in model (025 clinician 100+)   Improved patient-staff communication   - We also surveyed both doctors and nurses … there had been a substantial improvement in the efficiency and quality of communication with patients… nurses have said, we get less questions from families now (012 clinician teaching) |
| Patient experience | Knowledge that hospital takes patient needs seriously   - I think it was reassuring for them [patients] to know that what they felt and what mattered to them in their care was going to be captured and was going to be rolled out with staff and form the basis of what their care would look like. And so I think for patients it was very reassuring; that their needs were being taken very seriously (015 patient/family teaching) - [Patients knew] that staff at the hospital were listening to what really mattered to them and humanizing them (015 patient/family teaching) - The impact was that it was the patients language. It was their idea, we just helped to bring it to fruition; to ensure that it was not another document that would be just put up on a wall but something that everybody truly believed in (028 PE manager teaching)   Greater understanding of hospital instructions (due to new/improved resources)   - The discharge information sheets improved understanding for our patients and families… we could see how it provided clarity for the patient (027 PE manager <100) | Reassurance that hospital addresses what matters to patients   - We were delighted to see the progress that had been made and what had been developed and we could patients voices reflected in it… Everybody’s voice was heard and it was honoured (019 patient/family 100+) - When they [PE leads] came back with revisions to the patient handbook, you can see that they were paying attention; we’re not just putting time in and paying lip service to the issues you want to see… our comments were reflected in the finished product (023 patient/family 100+) - I saw my words and my voice in some of the documents that were created. I really did see my thoughts and my processes and my choices reflected in the product (029 patient/family 100+) - I’m very impressed with the final version because what they’ve done is they’ve also put in what we did at the back of the document which was putting contact information … most of our suggestions were taken and included in the document (035 patient/family <100)   Increased satisfaction with facilities, programs and services   - And it’s a substantial supper and a lighter lunch and inpatients seem to be very pleased with the transition (002 patient/family <100) - She [charge nurse] shared that it was because of the work that we had done earlier on in communication and planning that her patient had transitioned into this new location and was very happy (033 patient/family 100+)   Improved healthcare experience   - It’s just a much better working relationship with the nurses (007 patient/family teaching) - They really made big changes to dramatically improve the process and the communication between the hospital staff, family and patients … they’ve definitely made the kinds of improvements that I would have liked to have had when I was the person sitting in the hospital (033 patient/family 100+)   Greater understanding of hospital instructions (due to new/improved resources)   - I think the patient now gets a more concise and sensitive letter from the researcher with respect to explaining what it’s all about and it’s a two-pager versus sometimes there was 7 or 8 eight pages. And so they’ve done a very good job of making it much more readable and understandable to the patient and their caregivers (017 patient/family 100+) - So I think it [patient admission handbook] had a significant impact on patient admission as far as preparation and simplifying the process somewhat for the hospital; people are going in prepared with some knowledge (023 patient/family 100+) | Reassurance that hospital addresses what matters to patients   - Their [patient/family advisor] information is in that document. Their quotes are in that document. They knew it was reflective of what they said (040 clinician 100+)   Increased satisfaction with facilities, programs and services   - We have patient satisfaction rates of about 90% (006 clinician <100) - We saw an improvement in patient satisfaction (012 clinician teaching) - We’ve had lots of great feedback from families (022 clinician 100+) - So the patient satisfaction increases greatly from this [post-discharge contact program] (031 clinician 100+) - The impact for quality of care is increased patient satisfaction with their stay (037 clinician <100)   Improved healthcare experience   - Patients would say that their experiences are better following the changes that were made (025 clinician 100+) - I believe it did make the sessions [between healthcare workers and patients] more prudent (034 patient/family 100+)   Greater understanding of hospital instructions (due to new/improved resources)   - Patients were definitely in a sense in a better head space coming into the consultation sessions (034 patient/family 100+) |
|  |  |  |  |
| Patient outcomes | Decreased wait times   - So there’s been a significant reduction in the number of wait time hours between the Emergency Department to in-patient units (011 PE manager 100+)   Increased safety   - They’re rolling out elements of the model of care … So they’re spreading it unit by unit improving quality, safety, patient experience (011 PE manager 100+) | --- | Decreased falls   - Decreased risk for falls; we did see a decrease in falls (037 clinician <100)   Decreased readmission rates   - I’d say, we also saw a reduction in patient readmission rates as well (021 clinician 100+) - We measured the impact on readmission and we do see that it is a good return on investment. It [post-discharge contact program] does decrease patient readmissions (031 clinician 100+) |
